# Dissecting aortic aneurysm in Marfan syndrome is associated with losartan-sensitive transcriptomic modulation of aortic cells

**DOI:** 10.1172/jci.insight.168793

**Published:** 2023-05-22

**Authors:** Yifei Sun, Keiichi Asano, Lauriane Sedes, Anna Cantalupo, Jens Hansen, Ravi Iyengar, Martin J. Walsh, Francesco Ramirez

**Affiliations:** Department of Pharmacological Sciences, Icahn School of Medicine at Mount Sinai, New York, New York, USA.

**Keywords:** Genetics, Vascular Biology, Cardiovascular disease, Expression profiling, Extracellular matrix

## Abstract

To improve our limited understanding of the pathogenesis of thoracic aortic aneurysm (TAA) that leads to acute aortic dissection, single-cell RNA sequencing (scRNA-seq) was employed to profile disease-relevant transcriptomic changes of aortic cell populations in a well-characterized mouse model of the most commonly diagnosed form of Marfan syndrome (MFS). As result, 2 discrete subpopulations of aortic cells (SMC3 and EC4) were identified only in the aorta of *Fbn1^mgR/mgR^* mice. SMC3 cells highly express genes related to extracellular matrix formation and nitric oxide signaling, whereas the EC4 transcriptional profile is enriched in smooth muscle cell (SMC), fibroblast, and immune cell–related genes. Trajectory analysis predicted close phenotypic modulation between SMC3 and EC4, which were therefore analyzed together as a discrete MFS-modulated (MFSmod) subpopulation. In situ hybridization of diagnostic transcripts located MFSmod cells at the intima of *Fbn1^mgR/mgR^* aortas. Reference-based data set integration revealed transcriptomic similarity between MFSmod- and SMC-derived cell clusters modulated in human TAA. Consistent with the angiotensin II type I receptor (At1r) contribution to TAA development, MFSmod cells were absent in the aorta of *Fbn1^mgR/mgR^* mice treated with the At1r antagonist losartan. Altogether, our findings indicate that a discrete dynamic alteration of aortic cell identity is associated with dissecting TAA in MFS mice and increased risk of aortic dissection in MFS patients.

## Graphical abstract

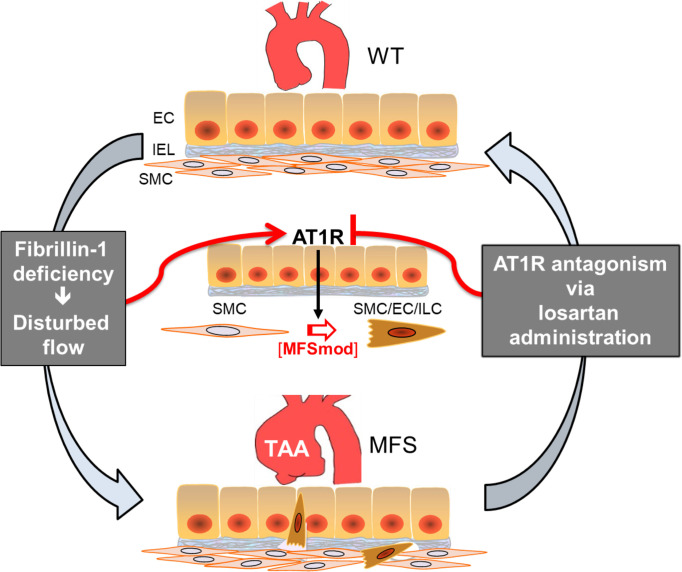

## Introduction

Marfan syndrome (MFS) is a relatively common connective tissue disorder caused by mutations in the extracellular matrix (ECM) protein fibrillin-1 that, by causing loss of tissue integrity, promote a variety of systemic manifestations, including thoracic aortic aneurysm (TAA) that leads to acute aortic dissection and rupture (1). Fibrillin-1 is widely expressed in the aorta as a component of the elastic fiber/microfibril network that separates and interconnects the individual layers of smooth muscle cells (SMCs) in the tunica media (2). Fibrillin-1 microfibrils also participate in anchoring the basal lamina underneath intimal endothelial cells (ECs) to the internal elastic lamina bordering the tunica media (2). Aortic disease in MFS is associated with endothelial dysfunction, SMC dedifferentiation and apoptosis, unproductive ECM remodeling, and localized inflammatory infiltrates (1). Although lifestyle modifications and antihypertensive drugs are routinely prescribed to slow TAA progression, once aortic diameter grows to 5 cm, prophylactic surgical repair remains the most effective treatment (1). Studies of mouse models of MFS have identified several disease-associated genes and signaling pathways (1, 3); however, there is still a need to integrate these disparate data with transcriptional contributions by different cell types.

The analysis of single-cell RNA sequencing (scRNA-seq) data has delineated gene expression profiles of aortic cells under a variety of conditions. This approach has revealed SMC heterogeneity within and between vascular beds for the expression of genes implicated in cell migration, adhesion, and inflammation (4). Likewise, aortic ECs were segregated into 3 distinct clusters based on enriched expression of genes associated with ECM production (EC1), lipid handling and angiogenesis (EC2), and lymphatic function (EC3) (5). Single-cell transcriptomic analyses have also documented that a small subset of vascular SMCs within atherosclerotic lesions acquire a transitional multipotent state that can differentiate into either plaque-stabilizing or plaque-destabilizing phenotypes (6, 7). Similarly, proatherogenic disturbed flow has been reported to reprogram gene expression of a small cluster of aortic ECs in a manner consistent with endothelial-mesenchymal cell transition and endothelial–immune cell–like transition (8). Recent studies have profiled the single-cell transcriptome of dilated aortas harvested from MFS mice with nondissecting TAA (*Fbn1^C1041G/+^* mice) and from MFS patients who underwent elective surgical repair (9, 10). Briefly, Pedroza et al. (9) identified a distinct cluster of SMCs modulated at the transcript level (modSMCs) only in aortic aneurysm tissue of *Fbn1^C1041G/+^* mice (9). These authors also reported that modSMCs cocluster with analogous cell subpopulations in an SMC lineage–traced atherosclerosis mouse model and in the dilated aortic root of an MFS patient (9). They further interpreted single-cell transcriptomic profiling at early and late stages of TAA progression to suggest a probable involvement of TGF-β signaling in driving mouse modSMCs (9). By contrast, a separate single-cell transcriptomic analysis of human aortic samples predicted impairment of TGF-β signaling by showing *TGFB1* gene upregulation in 2 clusters of adventitial fibroblasts in association with downregulation of components of TGF-β signal transducing pathways in all aortic cells (10).

Unlike most MFS patients, TAA in *Fbn1^C1041G/+^* mice develops very slowly and rarely progresses to acute aortic dissection, thus limiting the informative value of findings obtained with this mouse model of the human disorder (1, 3). The present study was therefore designed to delineate and validate disease-associated alterations of aortic SMC and EC transcriptomes in MFS mice that display fully penetrant dissecting TAA (*Fbn1^mgR/mgR^* mice) (11). Our major finding is the identification of an MFS-modulated aortic cell cluster (MFSmod) only in the *Fbn1^mgR/mgR^* aorta that is related at the transcript level to the SMC-derived modSMC cluster of the human TAA transcriptome (9). MFSmod cells are located in the intimal layer of the diseased aorta and disappear when *Fbn1^mgR/mgR^* mice are chronically treated with the antihypertensive drug losartan. Altogether, our findings indicate that MFSmod aortic cell identity is an important event associated with progressively severe arterial disease in both mice and humans.

## Results

### Identification of aneurysm-specific EC4 and SMC3 clusters.

**Figure 1:**
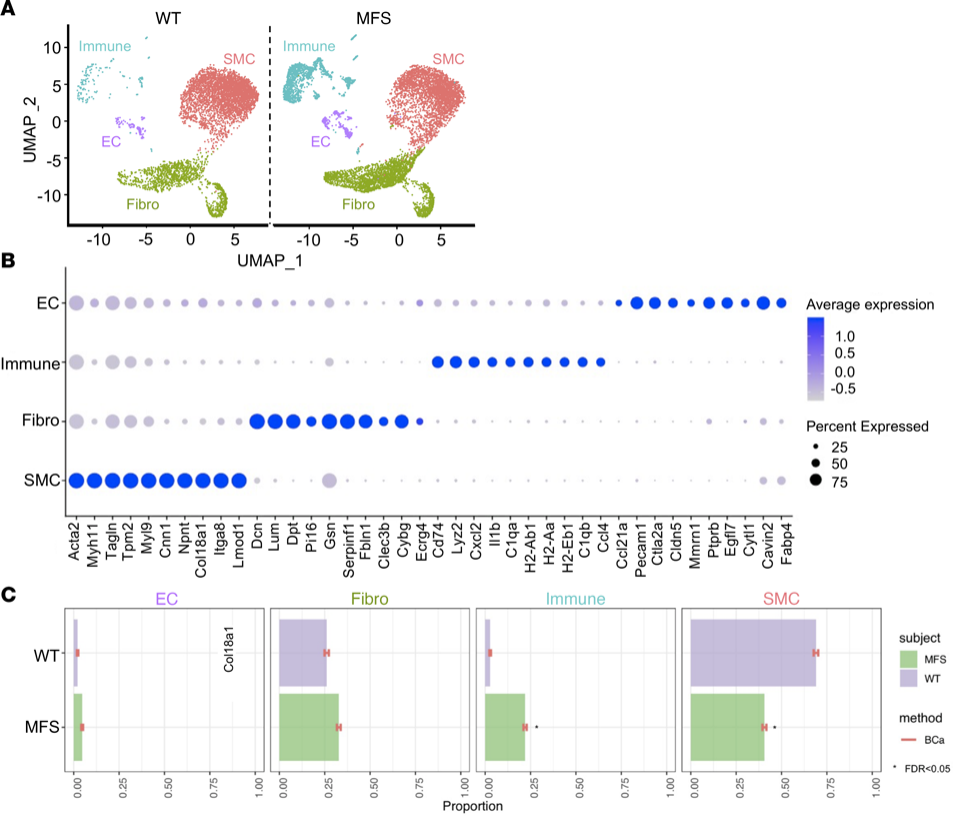
Transcriptomic atlas of ascending aorta cell types. (**A**) Uniform manifold approximation and projection (UMAP) dimensional reduction of individual cells recovered from *Fbn1^mgR/mgR^* mice (*n* = 3) and WT littermates (*n* = 3) shows 4 identified major aortic cell types classified based on their enriched genes and annotation predicted by singleR package in R along with marker gene identification. (**B**) Dot plot shows top 10 genes highly enriched in major aortic cell populations. Dot size corresponds to the percentage of cells expressing each gene, and dot color corresponds to the level of expression. (**C**) Relative proportion of major cell clusters in MFS and WT aortas was evaluated with scDC (v0.1.0) using bias-corrected and accelerated (BCa) bootstrap confidence intervals, and FDR significance (<0.05) between groups was calculated after GLM fitting (22).

TAA development has been extensively detailed in *Fbn1^mgR/mgR^* mice with regard to the rate of aneurysm growth, onset of elevated angiotensin II type I receptor (At1r) and TGF-β signaling, progression of aortic tissue degeneration, and timing of acute aortic dissection and wall rupture (11). Single-cell transcriptomic analyses of ascending thoracic aortas from *Fbn1^mgR/mgR^* mice and WT littermates were performed at around P45 when vessel dilation becomes statistically significant (Supplemental Figure 1; supplemental material available online with this article; https://doi.org/10.1172/jci.insight.168793DS1) and before onset of TGF-β–driven unproductive aortic tissue remodeling (11). Transmural aortic tissue was dissected from immediately above the aortic valve to immediately below the brachiocephalic artery, cleaned of contaminating tissues, and enzymatically digested. Single-cell suspensions were immediately processed for RNA-seq on a Chromium (10× Genomics). A total of 13,427 cells were recovered after quality control (including doublet cell analyses) and integrated for major cell type discovery. SMCs, ECs, fibroblasts, and immune cells are all major aortic cell types that were identified (Figure 1A). Expression of the top 10 marker genes selected for each population as described in the Methods confirmed their distinct gene signatures (Figure 1B). As expected, the relative proportions of SMCs and immune cells were smaller and larger in the mutant than the WT aorta, respectively (Figure 1C). Of note, there was also a modest increase in the proportion of ECs in *Fbn1^mgR/mgR^* aortas relative to the WT counterparts (Figure 1C).

**Figure 2:**
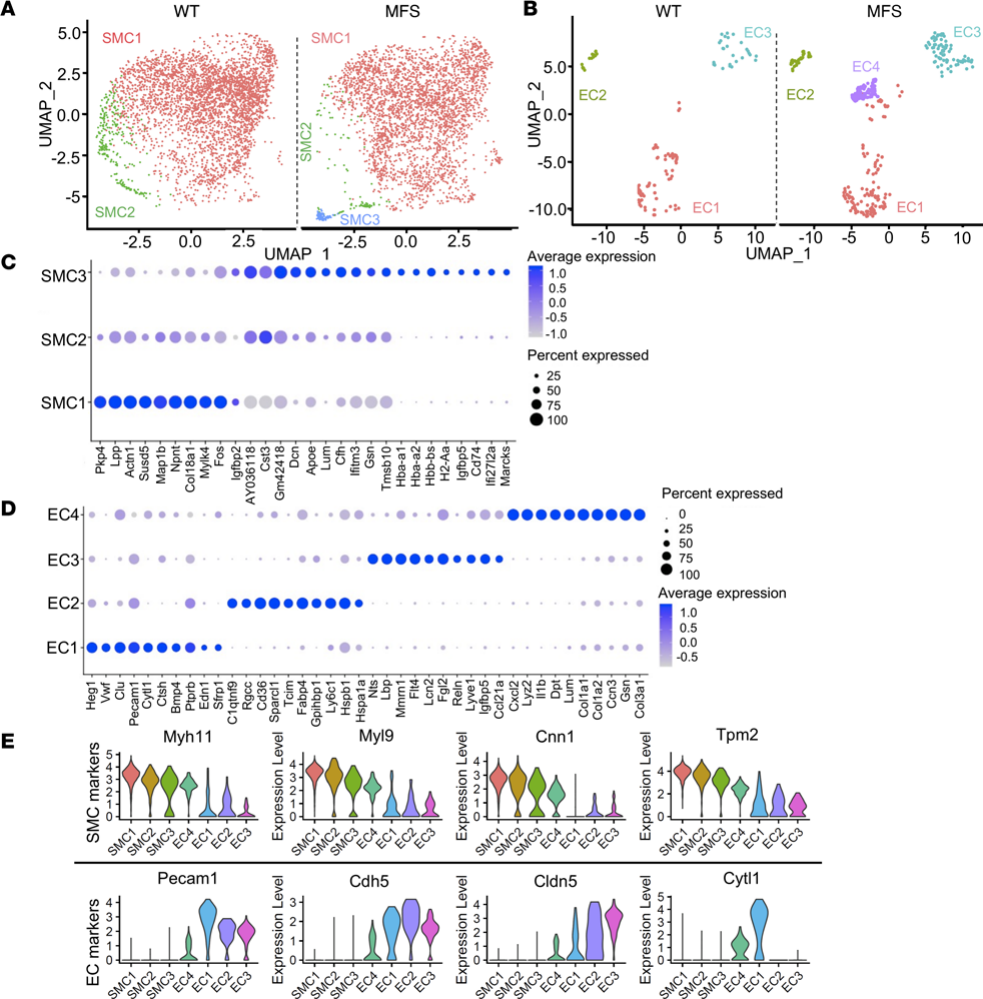
Disease-associated aortic cell types in MFS mice. Uniform manifold approximation and projection (UMAP) dimensional reduction of (**A**) SMC and (**B**) EC clusters extracted from the major cell populations. Dot plots showing top 10 genes highly enriched in SMC (**C**) and EC subpopulations (**D**). Dot size corresponds to the percentage of cells expressing each gene, and dot color corresponds to the level of expression. (**E**) Violin plots showing expression of SMC marker genes (upper) and EC marker genes (lower) in each cell subpopulation of *Fbn1^mgR/mgR^* aortas.

Consistent with the scope of our study, an unsupervised graph-based clustering segregated SMCs and ECs of the mutant and WT aortas into distinct subpopulations defined by the expression of the respective canonical markers (Figure 2). We found 3 distinct clusters of SMC subpopulations shared by WT and *Fbn1^mgR/mgR^* aortas (Figure 2A). SMC1 is the largest cluster of both mutant and WT samples and was enriched in the expression of genes coding for proteins of the contractile apparatus, e.g., actinin α1 (*Actn1*) and myosin light-chain kinase 4 (*Mylk4*) (Figure 2, C and E). SMC2 represents a discrete cell cluster that was substantially decreased in *Fbn1^mgR/mgR^* aortas and that displayed a transcriptomic profile in between SMC1 and SMC3 (Figure 2, C and E). Thus, SMC2 may conceivably represent a phenotypic transition between the SMC1 and SMC3 subclusters. SMC3 is another discrete cell cluster exclusively found in the *Fbn1^mgR/mgR^* aorta that is characterized by relatively low expression of contractility related genes and by augmented expression of genes related to the ECM, e.g., decorin (*Dcn*), lumican (*Lum*), and nitric oxide (NO) signaling (hemoglobin 1 [*Hba-a1*]) (Figure 2, C and E).

EC reclustering identified a total of 4 discrete subpopulations in WT and *Fbn1^mgR/mgR^* aortas; 3 of them (EC1–EC3) are common to both genotypes, whereas EC4 is only found in the fibrillin-1–deficient aorta (Figure 2B). EC1–EC3 correspond to the previously described endothelial clusters enriched in the expression of genes related to ECM production (EC1), lipid handling and angiogenesis (EC2), and lymphatic function (EC3) (Figure 2, D and E, and Supplemental Figure 2) (5). EC4 displays a transcriptional profile largely composed of the same genes previously shown to be upregulated in response to chronic disturbed flow (8), such as thrombospondin-1 (*Thbs1*) and marker genes of SMCs (myosin heavy chain 11 [*Myh11*], transgelin [*Tagln*], and smooth muscle 2 actin [*Acta2*]), fibroblasts (*Dcn* and *Lum*), and immune cells (histocompatibility 2, class II antigen [*Cd74*], antigen E1 [*H2-eb1*], antigen A [*H2-aa*], and antigen A1 [*H2-ab1*]) (Figure 2, D and E, and Supplemental Figure 3A). In line with this finding, en face tissue sections probed with antibodies against an indicator of EC polarization against flow, Golgi matrix protein 130 (GM130) (12), documented significantly disturbed flow in the aortas of *Fbn1^mgR/mgR^* mice (Supplemental Figure 3, B and C). Lastly, enrichment analysis of differentially expressed genes using the public database Reactome identified biological processes overrepresented in the various SMC and EC subpopulations, thus providing an additional characterization of these aortic cell types (Supplemental Figures 4 and 5). In particular, our analysis highlighted collagen and elastic fibrillogenesis, and integrin- and non–integrin-mediated cell-matrix interactions among the biological processes activated in the TAA-associated EC4 and SMC3 clusters, respectively (Supplemental Figures 4 and 5).

### Phenotypic modulation between SMC3 and EC4 clusters.

**Figure 3:**
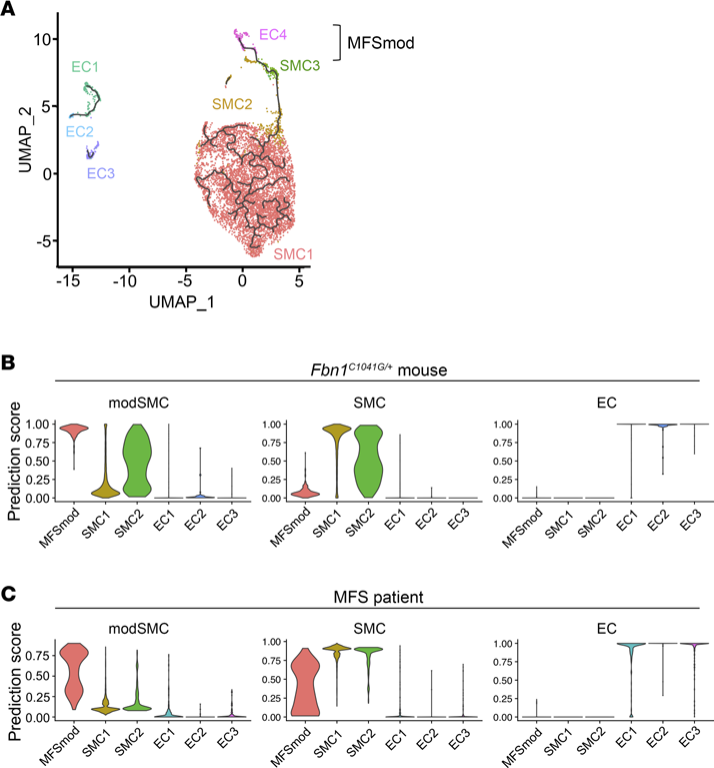
Transcriptomic similarities between modulated aortic cells in MFS mice and patient. (**A**) Trajectory analysis of EC and SMC subpopulations in both WT and *Fbn1^mgR/mgR^* samples indicating dynamic modulation between SMC3 and EC4, thus clustered together as MFSmod cells. (**B** and **C**) Violin plots showing the degree of transcriptomic homology of EC and SMC subpopulations in the ascending aorta of *Fbn1^mgR/mgR^* mice vs. either (**B**) the *Fbn1^C1041G/+^* aortic root/ascending aorta or (**C**) the aortic root of an MFS patient (9).

**Figure 4:**
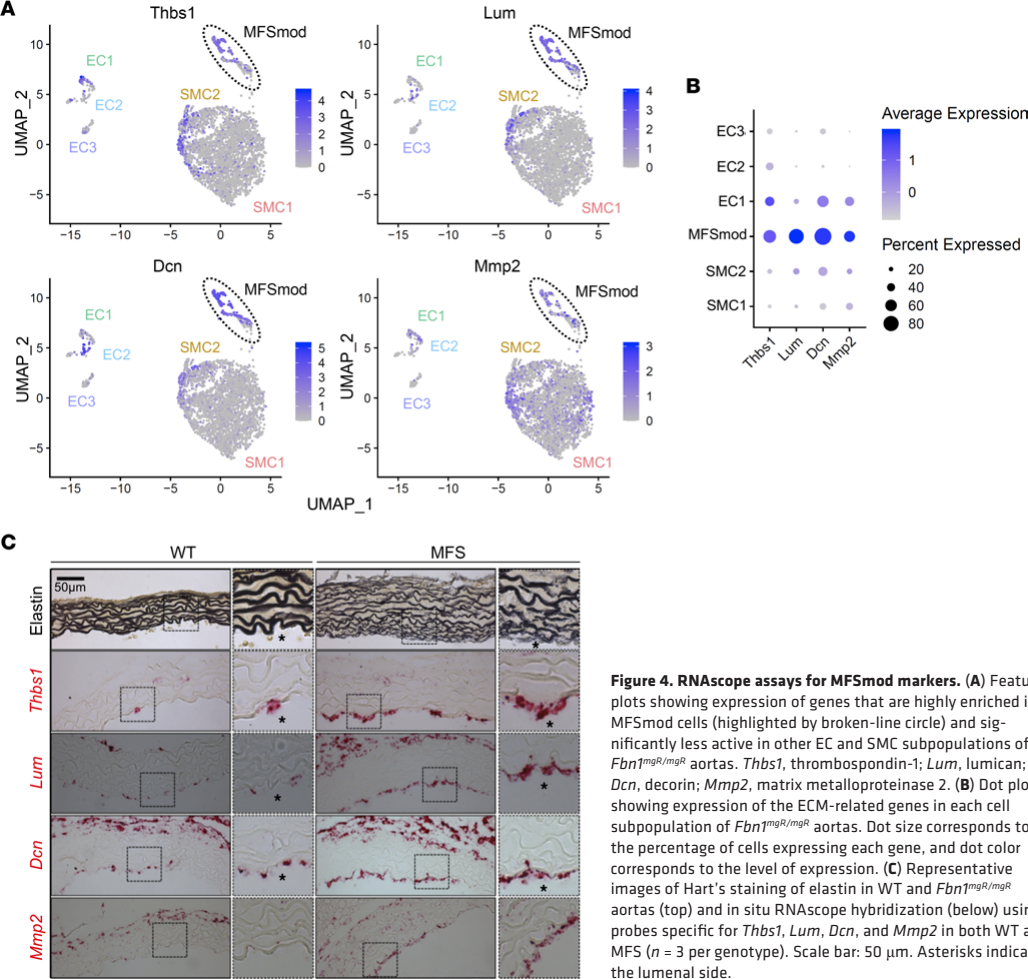
RNAscope assays for MFSmod markers. (**A**) Feature plots showing expression of genes that are highly enriched in MFSmod cells (highlighted by broken-line circle) and significantly less active in other EC and SMC subpopulations of *Fbn1^mgR/mgR^* aortas. *Thbs1*, thrombospondin-1; *Lum*, lumican; *Dcn*, decorin; *Mmp2*, matrix metalloproteinase 2. (**B**) Dot plots showing expression of the ECM-related genes in each cell subpopulation of *Fbn1^mgR/mgR^* aortas. Dot size corresponds to the percentage of cells expressing each gene, and dot color corresponds to the level of expression. (**C**) Representative images of Hart’s staining of elastin in WT and *Fbn1^mgR/mgR^* aortas (top) and in situ RNAscope hybridization (below) using probes specific for *Thbs1*, *Lum*, *Dcn*, and *Mmp2* in both WT and MFS (*n* = 3 per genotype). Scale bar: 50 μm. Asterisks indicate the lumenal side.

A doublet cell analysis using the DoubleFinder package predicted no enrichment of doublet clustering among the 133 EC4 cells and only 2 doublets out of the 127 SMC3 cells. We used trajectory analysis to investigate the possibility of dynamic transcriptomic modulations of ECs and SMCs in the ascending aorta of *Fbn1^mgR/mgR^* mice. As a result, the EC4 trajectory was placed closer to SMC3 than to any of the other EC clusters, thus predicting phenotypic dynamics between the SMC3 and EC4 subpopulations (Figure 3A). Furthermore, ligand-receptor analysis of scRNA-seq data revealed a higher degree of similarity between EC4 and SMC3 in both incoming and outgoing signaling pathways, compared with the other EC and SMC subpopulations (Supplemental Figure 6). This in silico analysis predicted that EC4 have elevated outgoing thrombospondin signaling, particularly the NO-signaling regulator Thbs1 (Supplemental Figure 7) (13). Based on these strong predictive findings, subsequent analyses were performed by combining the SMC3 and EC4 transcriptomes into a single modulated cell cluster (MFSmod) exclusively associated with dissecting TAA (Figure 3A). RNAscope in situ hybridization using probes against transcripts from ECM-related genes, which are expressed at lower levels in cell clusters other than MFSmod (Figure 4, A and B), revealed a patchy distribution of this unique subpopulation in the intimal cell layer of the MFS aorta (Figure 4C and Supplemental Figure 8). Immunofluorescent costaining of en face tissue sections confirmed MFSmod localization by identifying cells that coexpress endothelial CD31 and EC4 unique marker Thbs1 (Supplemental Figure 9). *Thbs1* expression in the intimal layer implicitly validated the computational prediction of elevated EC4 secretion of this matricellular protein (Figure 4C and Supplemental Figure 7). As expected, adventitial fibroblasts were also found to express some of these ECM-related genes (Figure 4C).

### TAA-associated aortic cell modulation occurs in both MFS mice and MFS patients.

Next, we compared the aortic EC and SMC transcriptomes of *Fbn1^mgR/mgR^* mice, including the MFSmod transcriptome, with those that Pedroza et al. (9) recently identified in aneurysmal tissues of *Fbn1^C1041G/+^* mice and an MFS patient. Specifically, the authors identified a distinct cluster of SMCs modulated at the transcript level (modSMCs) only in aortic aneurysm tissue of *Fbn1^C1041G/+^* mice (9). They also reported that modSMCs cocluster with analogous cell subpopulations in an SMC lineage–traced atherosclerosis mouse model, and in the dilated aortic root of an MFS patient (9). Integration of scRNA-seq data sets showed that major aortic cell types (ECs, SMCs, fibroblasts, and immune cells) of *Fbn1^mgR/mgR^* and *Fbn1^C1041G/+^* mice cluster together, and that MFSmod cells reside closely to and slightly overlap with the modSMC subpopulation (Supplemental Figure 10). Additional comparative analyses predicted close transcriptomic homology between MFSmod and both mouse and human modSMCs (Figure 3, B and C). Lastly, highly expressed MFSmod genes overlapping with mouse and human modSMCs were identified and found to largely correspond to biological pathways related to ECM remodeling and cell-matrix interactions (Supplemental Figure 11, A and B).

As enriched TGF-β1 signaling was implicated in driving modSMCs in *Fbn1^C1041G/+^* mice, we used the same computational approach as Pedroza et al. (9) to predict whether or not TAA-associated phenotypic cell modulation was driven by TGF-β in *Fbn1^mgR/mgR^* mice as well. Even though our analysis predicts *Tgfb2* and *Tgfb3* upregulation in SMCs of P45 *Fbn1^mgR/mgR^* mice, concomitant elevation of *Smad7* expression in these cells suggests inhibition rather than activation of the canonical TGF-β signaling pathway (Supplemental Figure 12A). Furthermore, our findings indicate that upregulation of TGF-β ligands and inhibitory Smad7 is largely associated with the SMC1 cluster rather than MFSmod cells (Supplemental Figure 12B). We therefore conclude that enhanced TGF-β signaling is not a prominent upstream driver of aortic cell phenotypic modulation in MFS.

### Losartan-mediated TAA mitigation is associated with MFSmod disappearance.

**Figure 5:**
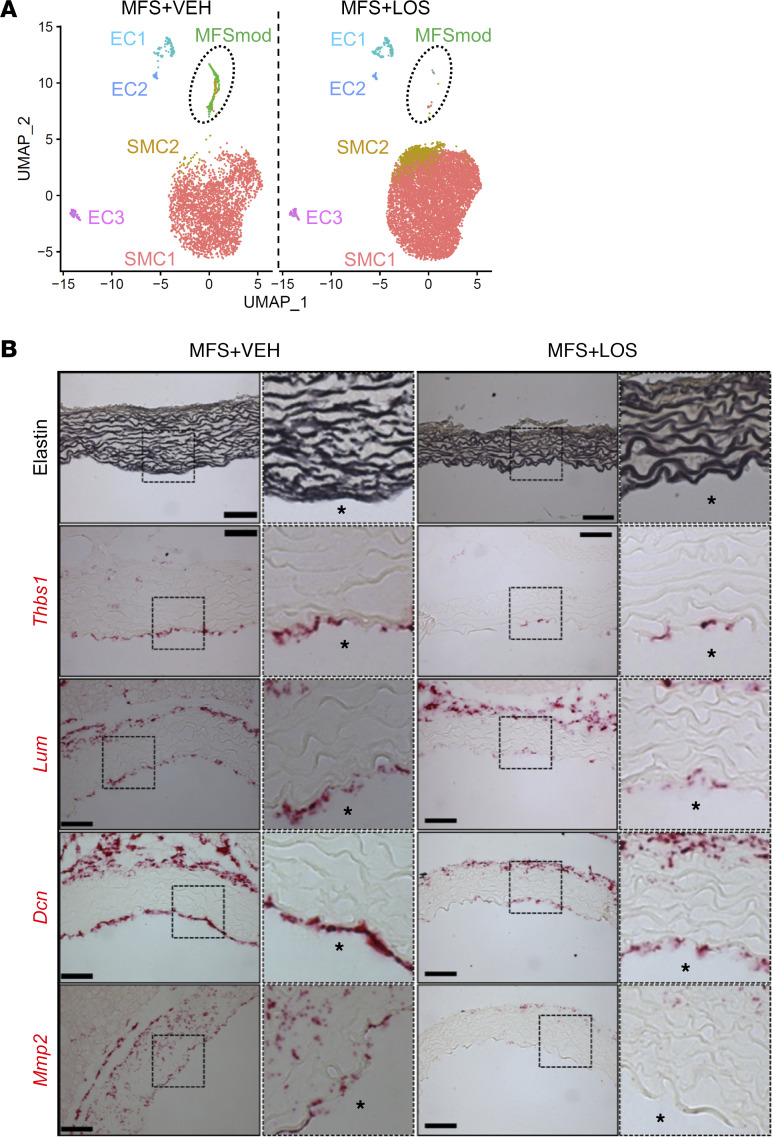
MFSmod absence in losartan-treated mutant mice. (**A**) Uniform manifold approximation and projection (UMAP) showing aortic EC and SMC subpopulations of *Fbn1^mgR/mgR^* mice treated with vehicle (VEH; *n* = 3) or losartan (LOS; *n* = 4). Broken-line circle indicates unique subpopulations of the mutant aorta. (**B**) Illustrative images of Hart’s staining of elastin (top) and in situ RNAscope hybridization (below) using probes specific for *Thbs1*, *Lum*, *Dcn*, and *Mmp2* in the aortas of losartan-treated and untreated *Fbn1^mgR/mgR^* mice (*n* = 3 per group). Scale bars: 50 μm. Asterisks indicate the lumenal side.

Inhibition of At1r signaling through systemic administration of losartan substantially mitigates TAA progression in different mouse models of MFS (3). We therefore performed scRNA-seq experiments to assess whether the beneficial action of At1r antagonism may be associated with transcriptomic changes in the *Fbn1^mgR/mgR^* aorta. Drug effectiveness was ensured by monitoring aortic diameter size at the end of experimental time, i.e., after 4 weeks of chronic drug treatment (Supplemental Figure 1). A total of 20,783 cells were recovered after quality control and log normalization, and employed for each treatment condition. Data from WT and losartan- or vehicle-treated *Fbn1^mgR/mgR^* mice were integrated before SMC and EC reclustering and analysis. Losartan treatment normalized the transcriptomic cell profile of the ascending thoracic aorta by increasing SMC1 and SMC2 relative representation and by inhibiting MFSmod formation (Figure 5A). RNAscope in situ hybridization provided in vivo validation that these transcriptomic changes were associated with downregulation of the 4 ECM-related genes highly expressed in the MFSmod cell cluster of *Fbn1^mgR/mgR^* mice (Figure 5B). These findings further reiterate that MFSmod is specifically associated with arterial disease.

## Discussion

By impairing the structural integrity of the vessel wall, mutations in fibrillin-1 trigger a cascade of cellular and molecular abnormalities in responses to physiological hemodynamic load that promote progressive enlargement of the ascending thoracic aorta, leading to acute aortic dissection (1–3). A large body of work has implicated dysregulated angiotensin II, NO, and TGF-β signaling as major molecular determinants of TAA progression in MFS mouse models of dissecting and nondissecting aneurysm (*Fbn1^mgR/mgR^* and *Fbn1^C1041G/+^* mice, respectively) (3). The major finding of our single-cell transcriptomic profiling of aortic cells in *Fbn1^mgR/mgR^* mice is the identification of a discrete subpopulation of phenotypically modulated cells (MFSmod cells) that are (a) uniquely associated with dissecting TAA, (b) enriched in the expression of disturbed flow–related genes, (c) sensitive to the action of an At1r antagonist that delays TAA progression, and (d) similar at the transcript level to an SMC modulated cell cluster previously identified in human aneurysmal tissue (9).

Interpretation of data from single-cell transcriptomic profiling at early and late stages of TAA progression recently correlated upregulation of *Klf4* and TGF-β–responsive genes with driving SMC modulation in the aorta of *Fbn1^C1041G/+^* mice (9). Additional bioinformatics analyses coclustered modSMCs of *Fbn1^C1041G/+^* mice with analogous cell subpopulations in an SMC lineage–traced atherosclerosis mouse model, and in the dilated aortic root of an MFS patient (9). Our identification of TAA-associated MFSmod in the aorta of *Fbn1^mgR/mgR^* mice was based on the finding of 2 discrete aortic cell clusters (SMC3 and EC4) that a pseudotime trajectory analysis placed closer to each other than to any other SMC and EC subpopulation. SMC3 is characterized by the relative upregulation of genes related to the ECM and NO signaling, and the relative downregulation of genes coding for components of the contractile network. The EC4 cluster, on the other hand, displays a hybrid transcriptomic profile closely resembling the mesenchyme/immune cell expression pattern recently reported to characterize ECs reprogrammed to proatherogenic phenotypes by disturbed flow (8). We speculate that disturbed flow may also reprogram aortic SMCs to undergo mesenchymal-endothelial transition (MEndT) in *Fbn1^mgR/mgR^* mice. Our conclusion is based on the following indirect evidence: (a) MFSmod cells are of SMC origin because of their transcriptomic similarity to modSMCs of *Fbn1^C1041G/+^* mice, which is in turn analogous to an SMC lineage–traced cell subpopulation in atherosclerotic mice; and (b) SMC-derived MFSmod cells include subcluster EC4, which express canonical endothelial marker genes.

MEndT is a rare phenomenon that was also reported to occur after acute ischemic heart injury when cardiac fibroblasts participate in neovascularization by adopting an EC-like phenotype (14). An important feature that distinguishes TAA-associated MEndT from cardiac hypertrophy–associated MEndT is the identity of the mesenchymal cell type (SMCs vs. fibroblasts). Additionally, whereas cardiac MEndT plays a protective role, a strict MFSmod association with TAA formation conceivably reflects a pathogenic contribution of this cell subpopulation. For example, MFSmod cell localization in the intimal layer could be interpreted to reflect flow-induced reprogramming of discrete subendothelial SMCs to breach the internal elastic lamina. We previously documented a comparable mitigation of TAA pathology in *Fbn1^mgR/mgR^* mice either treated with losartan or lacking At1r signaling in ECs (11, 15). Thus, we speculate that the MFSmod association with losartan-induced TAA mitigation implicates At1r signaling in ECs in mediating flow-induced reprogramming of this discrete cluster of subendothelial cells. Ongoing investigations are characterizing the activating mechanism(s) and possible pathogenic contribution(s) of MFSmod.

Pedroza et al. (9) interpreted the finding of high expression of *Tgfb1* and potential genomic targets of TGF-β signaling in modSMCs to suggest the cytokine’s involvement in driving this phenotypic modulation in the *Fbn1^C1041G/+^* aorta (9). However, we found no evidence of augmented expression of genes related to the canonical TGF-β signaling pathway in the MFSmod transcriptome. In point of fact, upregulation of both TGF-β1 and TGF-β2 ligands and inhibitory Smad7 in the large SMC1 cluster indicates a potential block of TGF-β signaling in the media of P45 *Fbn1^mgR/mgR^* mice. Such a conclusion is in line with the report of potentially compromised TGF-β signaling in aneurysmal aortic tissue of MFS patients (10). It is also consistent with our earlier findings that Smad2 phosphorylation levels in the aorta of *Fbn1^mgR/mgR^* mice increase at a later stage than P45, and that systemic TGF-β neutralization between P16 and P45 dramatically accelerates the incidence of acute aortic dissection and rupture (11). Substantial differences in TAA natural history are likely to account for this apparent discrepancy between the MFS mouse models used in our and Pedroza et al.’s (9) studies.

We acknowledge certain limitations for using scRNA-seq as it may pertain to our comprehensive analysis, whereby aspects of low coverage and depth of sequence complexity, and inability to capture the entire spectrum of cells and transcript isoforms may fail to delineate all biological processes underlying TAA development. The efficient isolation of cell numbers and their proportion are additional technical challenges, particularly when studying a mouse model of early-onset, progressively severe aneurysm. In spite of these limitations, our efforts nonetheless point to overwhelming evidence for discrete EC and SMC subpopulations linked to the disease phenotype and thus, provide a strong roadmap for future investigations supported by advances in single-cell genomic technologies.

## Methods

### Mice.

Hypomorphic *Fbn1^mgR/mgR^* mice were routinely backcrossed for 10 generations on the C57BL/6J background (Jackson Laboratory, strain 00664) to avoid possible phenotypic changes due to genetic drift (11). Mutant mice and WT littermates were euthanized to harvest aortic specimens spanning from immediately above the aortic root to immediately before the brachiocephalic artery. Drug treatment included *Fbn1^mgR/mgR^* mice that received either losartan (0.6 g/L; LKT Labs) or vehicle through drinking water from P16 to P45, as well as vehicle-treated WT mice (11). Ascending aorta diameters were monitored by echocardiography at the end of treatment; based on these data, selected mutant mice were euthanized for ex vivo analyses (11). Vessel diameters were measured by a VisualSonics Vevo 2100 imaging system equipped with a 40-MHz transducer. Long-axis parasternal views of the aorta were captured using B-mode under isoflurane anesthesia, and 4 images per mouse were measured and averaged to obtain diameter (mm) values (11). All experiments were performed using male mice to ensure consistency with our previous studies of cardiovascular abnormalities in *Fbn1^mgR/mgR^* mice (3) by avoiding possible sex-related variances.

### En face immunostaining.

Aortas harvested from euthanized WT and *Fbn1^mgR/mgR^* mice (*n* = 3 per genotype) were perfused with PBS and 4% paraformaldehyde and cut open longitudinally (en face), as described previously (16). Samples were fixed in 4% paraformaldehyde for 30 minutes at room temperature followed by incubation with permeabilization/blocking buffer (2.5% BSA, 5% milk, and 0.5% Triton X-100 in PBS) for 1 hour. Aortic en face preparations were incubated overnight at 4**°**C with either 1:100 dilution of primary antibody against Golgi matrix protein 130 (GM130; 610822, BD Biosciences) or with 1:50 dilution of primary antibodies against CD31 (553370, BD Biosciences) and Thbs1 (39-9300, Thermo Fisher Scientific) followed by incubation with fluorophore-conjugated secondary antibody at room temperature for 1 hour and DAPI counter staining. Fluorescence images were acquired using an LSM880 confocal microscope (Zeiss) with 63× (GM130 staining) or 40× and 100× (CD31 and Thbs1 double staining) objectives; *Z*-stack images were overlaid and exported by ZEN Blue software (Zeiss). Flow-induced EC polarization was evaluated as the angle between the vector of flow direction (obtained by knowledge of flow direction within the slide) and the line from the center of the nucleus to the center of the Golgi. To this end, 5 different aortic fields per mouse were imaged and independently analyzed by 2 individuals blinded to genotype. Cells were classified according to their orientation against, alongside, and oblique to the direction of blood flow, and the cumulative estimates for each grouping are expressed as percentage over the total cell number counted per field (12). Values per mouse were averaged and plotted using GraphPad Prism software 9.0.

### In situ hybridization.

Aortic specimens (*n* = 3 per genotype and treatment) were fixed with formalin, embedded in paraffin, and sectioned at 5 μm thickness. Hart’s staining was used to evaluate elastic architecture (17). The RNAscope 2.5 HD RED system (Advanced Cell Diagnostics [ACD]) was used to localize gene transcripts in situ according to the manufacturer’s instructions. Probes included those against *Thbs1* (457891, ACD), *Lum* (480361, ACD), *Dcn* (413281, ACD), and *Mmp2* (315931, ACD). Brightfield images were acquired using a Leica microscope (model DM2500) mounted with 10× and 40× objectives.

### scRNA-seq.

Ascending thoracic aortas were harvested from euthanized P45 *Fbn1^mgR/mgR^* and WT mice (*n* = 3 per genotype), and losartan- and vehicle-treated *Fbn1^mgR/mgR^* mice (*n* = 4 and 3, respectively). Aortic tissues of each genotype and treatment arm were pooled together, cleaned, minced, and digested in Hank’s balanced salt solution (14170112, Gibco) containing elastase (0.5 mg/mL, LS006365, Worthington) and collagenase A (10103578001, Roche) for 1 hour at 37°C and then passed through a 40 μm filter. Single-cell suspensions were incubated with eBioscience 1× RBC Lysis Buffer (00-4333-57, Invitrogen) for 3 minutes and collected in PBS containing 1% UltraPure BSA (AM2616, Invitrogen). After confirming cell count and viability using an EVOS M7000 imaging system (Invitrogen), each sample was immediately processed by the Genomics Core at Icahn School of Medicine at Mount Sinai according to the manufacturer’s instructions (Chromium Next GEM Single Cell 3′ Reagent Kits v3.1, 10× Genomics).

### scRNA-seq data processing and analysis.

Initial sequence alignment was performed using CellRanger (18). Analysis Pipeline (10× Genomics) and GRCm38 (mm10) genome assembly (https://support.10xgenomics.com/single-cell-gene-expression/software/release-notes/build#mm10_2020A) were used as the reference genome throughout our analysis. Sequencing data were examined using Seurat package (v4.0.6) under R (v4.1.1.) (19). Cells with greater than 10% expression of mitochondrial genes and cells with expression of less than 600 or greater than 5000 genes were filtered out from further analysis. Each sample was preprocessed individually before being integrated by group type. Doublet cell proportion was estimated using the DoubleFinder package with threshold nFeature>200, mitochondria percentage<10 (20). Optimal pK value was selected based on cell clustering. Doublet cell number was predicted based on Poisson statistics with and without homotypic doublet proportion. Log normalization and canonical correlation analysis methods were applied to discover anchors among samples. Iterative pairwise integration was used to define the distance between data sets, which was used to cluster the distance matrix. Once integrated, significant principal components were used to perform unsupervised K-nearest neighbor (KNN) graph-based clustering. Major aortic cell types were identified based on the expression of canonical marker genes and the result from the singleR automatic annotation method using its built-in references (21). To analyze individual cell types, cell subsets from those annotations were renormalized and reintegrated for cell type subcluster calls generated as uniform manifold approximation and projection (UMAP). Identification of marker genes of each cluster/subcluster relied on differential expression analysis (Wilcoxon’s rank-sum test) of FindAllMarkers function of Seurat (https://satijalab.org/seurat/reference/findallmarkers) with the requirement of 25% expression in each given cluster/subcluster and an FDR cutoff of less than 0.05 and log_2_(fold change) greater than 0.25. Monocle 3 was used for trajectory analysis on selected groups of cells (22). After log normalization and running PCA, batch correction and dimension reduction were performed for cell clustering and assigning partitions. Reversed graph embedding was used to learn the principal graph from the reduced dimension space, and then the individual position of every single cell was plotted in a learned trajectory. CellChat (v1.1.3) was employed to quantitatively infer and analyze possible intercellular communications (23). Overexpressed ligand and receptor interactions were identified for each population, and resulting data were subsequently projected onto a protein-protein interaction network. Communication patterns were inferred by assigning each interaction a probability and performing a permutation test, and communication probability was calculated by modeling ligand-receptor–mediated interactions using the law of mass. *Fbn1^C1041G/+^* and *Fbn1^mgR/mgR^* data set integration, analysis, and comparison were performed by Seurat using the SCTransform integration. Marker genes for modSMCs and the MFS patient were built with the FindMarkers function via the nonparametric Wilcoxon’s rank-sum test, with the requirement of minimum percentage greater than 0.25 and log2(fold change) greater than 0.25. Data sets of the MFS patient (GSM4646673) and *Fbn1^C1041G/+^* mouse (GSM4646669 and GSM4646670) were downloaded from the NCBI Gene Expression Omnibus (GEO GSE153534) (9). Enriched pathways in each unique subpopulation were predicted using the Reactome package (https://bioconductor.org/packages/release/bioc/html/ReactomePA.html) based on their marker genes. Benjamini-Hochberg correction was applied to adjust for multiple comparisons.

### Data sharing and access.

FASTQ data used for the present study, including MFS mice with and without losartan treatment and respective WT littermates, are publicly available via the NCBI (GEO GSE227776).

### Statistics.

Marker genes for each cluster were identified using FindAllMarkers function of the Seurat package (Wilcoxon’s rank-sum test with the requirement of >25% expression, FDR cutoff < 0.05, and log_2_[fold change] > 0.25). Relative proportion of major cell groups was evaluated with scDC (v0.1.0) (https://github.com/SydneyBioX/scDC) using bias-corrected and accelerated (BCa) bootstrap confidence intervals, and FDR significance (<0.05) between groups was calculated after generalized linear model (GLM) fitting (22). For cell-cell communication analysis, the trimean method was used to compute the communication probability between any interacting cell groups. Communication probability on the signaling-pathway level was computed with a *P* value threshold of 0.05. Cell-cell communication was filtered out when fewer than 10 cells were observed in certain cell groups. For evaluation of EC polarization, logit transformation was performed for percentage of each EC orientation prior to 1-way ANOVA test (*P ≤ 0.05); data are presented using GraphPad Prism software 9.0 as mean ± SEM. Aortic diameters obtained by ultrasound were evaluated with 1-way ANOVA followed by Tukey’s post hoc analysis or Bonferroni’s test using GraphPad Prism software.

### Study approval.

The Institutional Animal Care and Use Committee of the Icahn School of Medicine at Mount Sinai in New York City reviewed and approved all animal studies.

## Author contributions

YS and KA worked closely together in designing, performing, and interpreting all the scRNA-seq experiments, with the latter individual devoting more time to the experimental aspect of the study under FR’s supervision, and the former on the computational analyses under MJW’s supervision. LS and AC performed in vivo validation experiments of disturbed flow and MFSmod tissue localization. JH provided independent evaluation of the computational findings under RI’s supervision. FR conceived the study and wrote the manuscript, with critical input from all authors.

## Supplementary Material

Supplemental data

## Version 1 04/06/2023

In-Press Preview

## Version 2 05/22/2023

Electronic publication
