# Supplementary material for: Dissecting aortic aneurysm in Marfan syndrome is associated with losartan-sensitive transcriptomic modulation of aortic cells
Source: JCI Insight. 2023 May 22;8(10):e168793. doi: 10.1172/jci.insight.168793 (PMC10322683; doi:10.1172/jci.insight.168793)
Supplement: Supplemental data [file jciinsight-8-168793-s114.pdf]

Supplemental Material

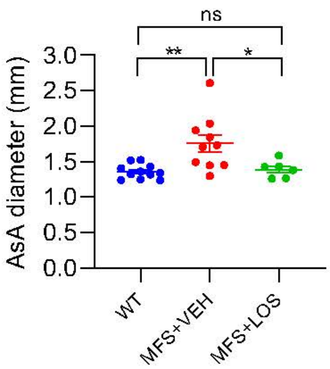

Comparison	Summary	Adjusted *p* value	Post hoc analysis
WT vs. MFS+VEH	**	0.0035	Tukey's multiple comparisons test
WT vs. MFS+LOS	ns	0.964	II
MFS+VEH vs. MFS+LOS	*	0.024	II
WT vs. MFS+VEH	**	0.0038	Bonferroni multiple comparisons test
WT vs. MFS+LOS	ns	> 0.9999	II
MFS+VEH vs. MFS+LOS	*	0.0275	II

Figure S1

Dot plots showing thoracic aorta diameters of P45 WT and *Fbn1*$^{mgR/mgR}$ mice treated with vehicle (MFS+VEH) or losartan (MFS+LOS). Asterisks signify statistical significances at *$p \leq 0.05$ and **$p \leq 0.01$. The table below summarizes the statistical analyses of the echocardiographic data.

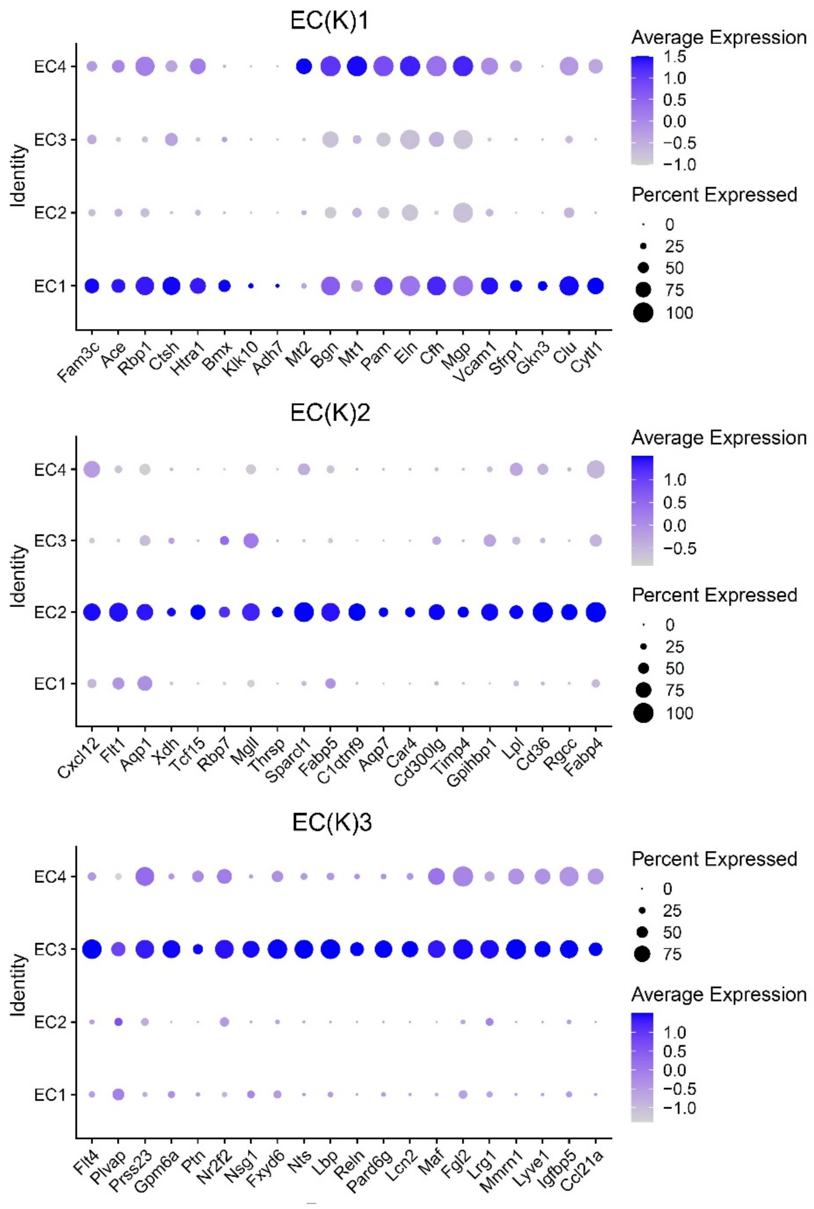

Figure S2

Dot plots showing transcriptional homology between the EC clusters identified in WT mice by Kalluri et al. (5) (EC(K)1-3) and those we identified in *Fbn1*$^{mgR/mgR}$ mice. Dot size corresponds percentage of cells expressing each gene, and dot color corresponds to the level of expressions.

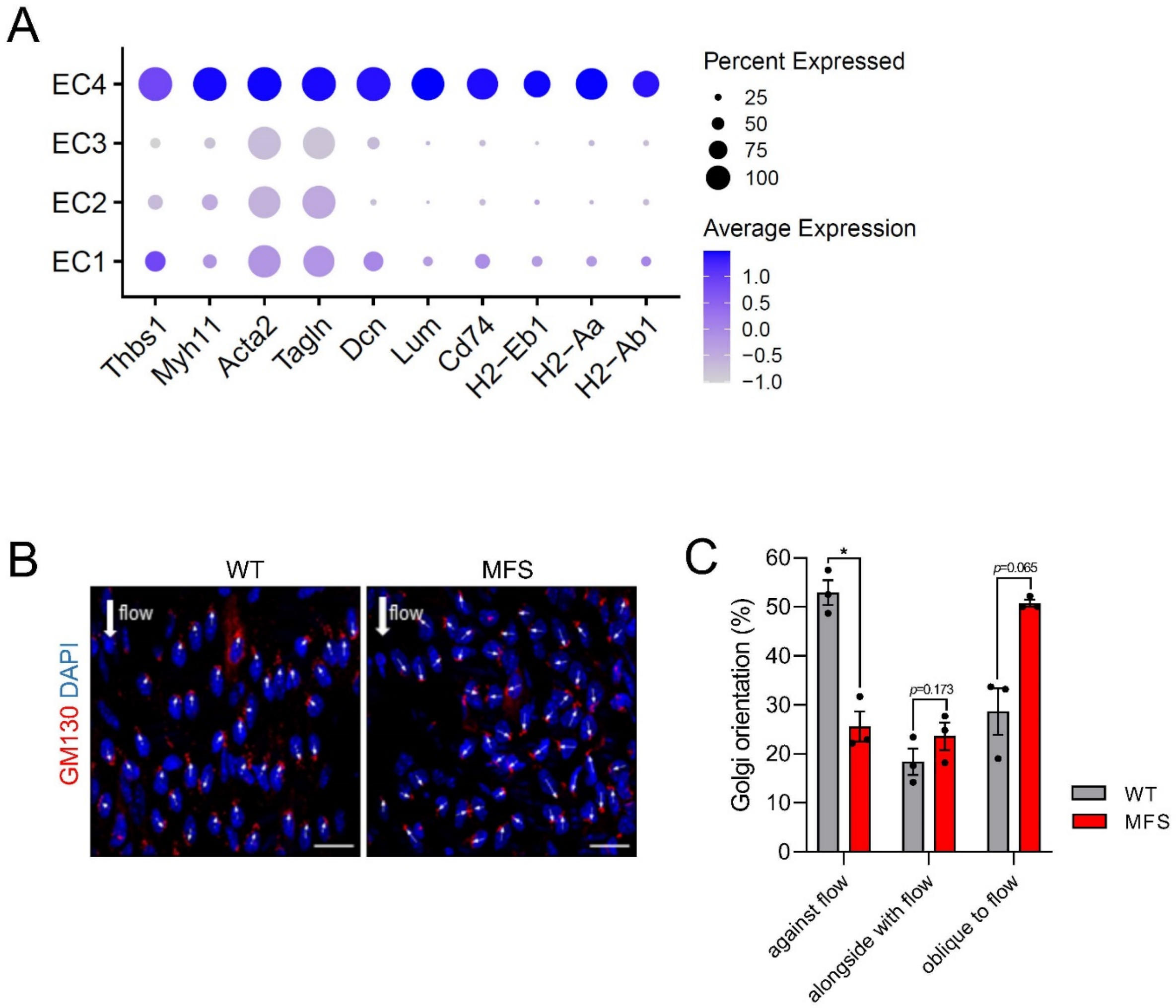

Figure S3

(A) Dot plot showing expression of genes in EC clusters of *Fbn1*$^{mgR/mgR}$ aortas that were previously shown to be upregulated in response to chronic disturbed flow (8). Dot size corresponds percentage of cells expressing each gene, and dot color corresponds to the level of expressions. **(B)** Illustrative images of *en face* aortic tissue sections from WT and *Fbn1*$^{mgR/mgR}$ (MFS) mice probed with GM130 antibody (red) and counter-stained with DAPI (blue); large white arrow indicates the direction of flow, whereas the thinner one points to the position of the Golgi apparatus relative to the nucleus in EC of WT and *Fbn1*$^{mgR/mgR}$ aortas. **(C)** Bar graphs summarize the percentage of cells with different Golgi orientations in the WT and *Fbn1*$^{mgR/mgR}$ samples (n= 3 per genotype). Scale bar = 20 µm. Data are presented as mean ±SEM; the asterisk indicates a statistically significant difference determined by logit transformation followed by one-way ANOVA (*$p \leq 0.05$).

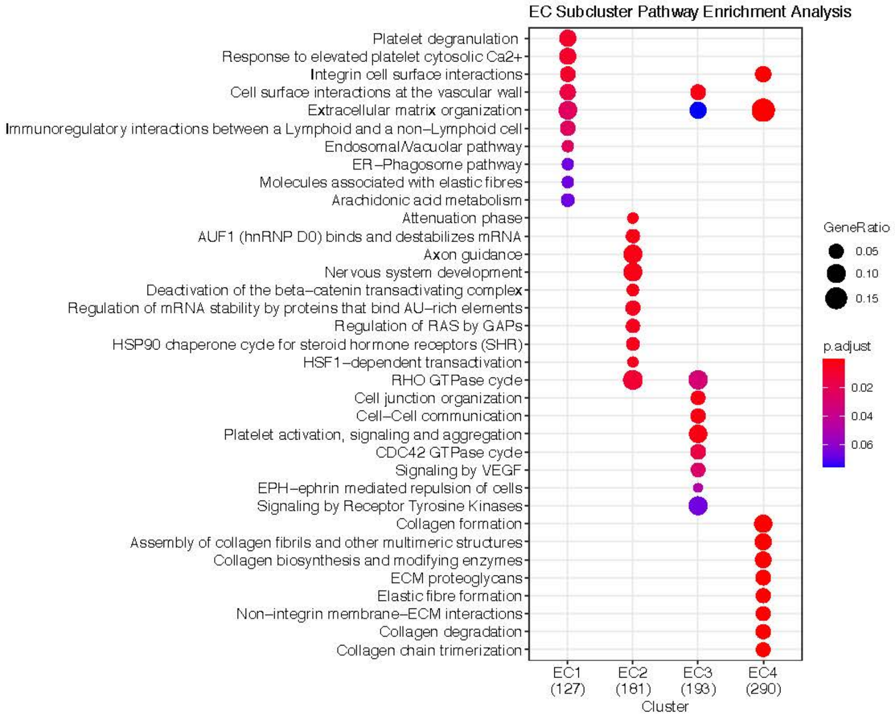

Figure S4

Dot plots of top 10 biological pathways enriched in differentially expressed genes of indicated endothelial cell (EC) clusters.

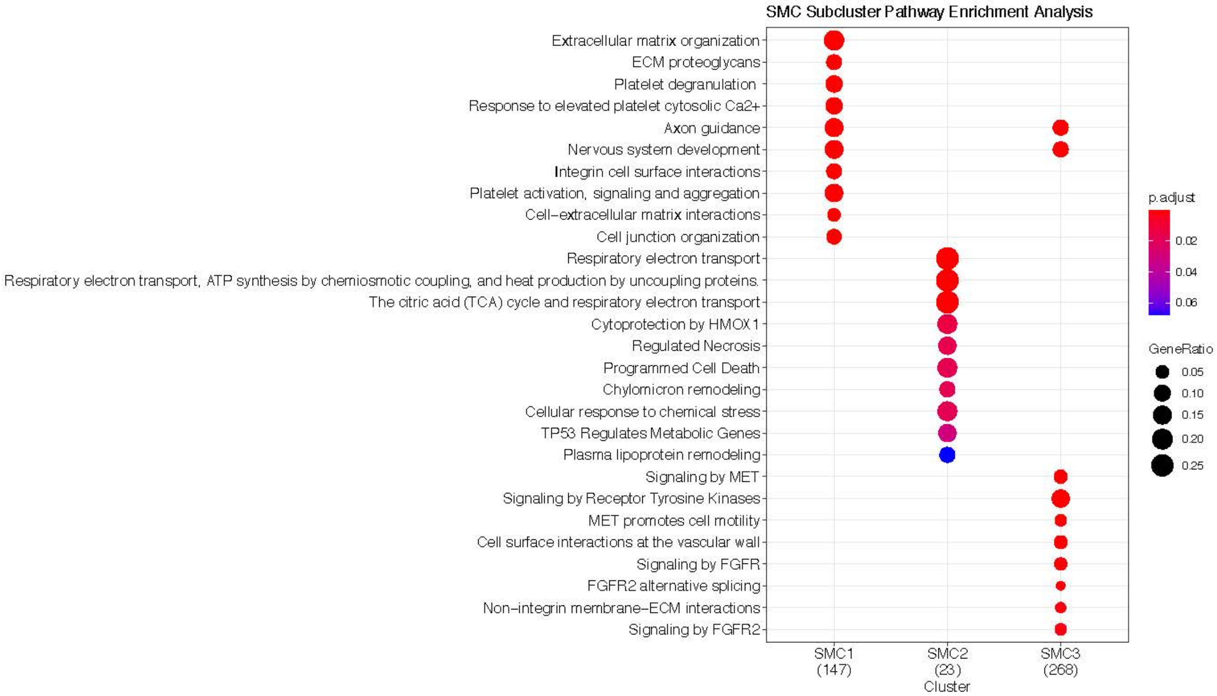

Figure S5

Dot plots of top 10 biological pathways enriched in differentially expressed genes of indicated smooth muscle cell (SMC) clusters.

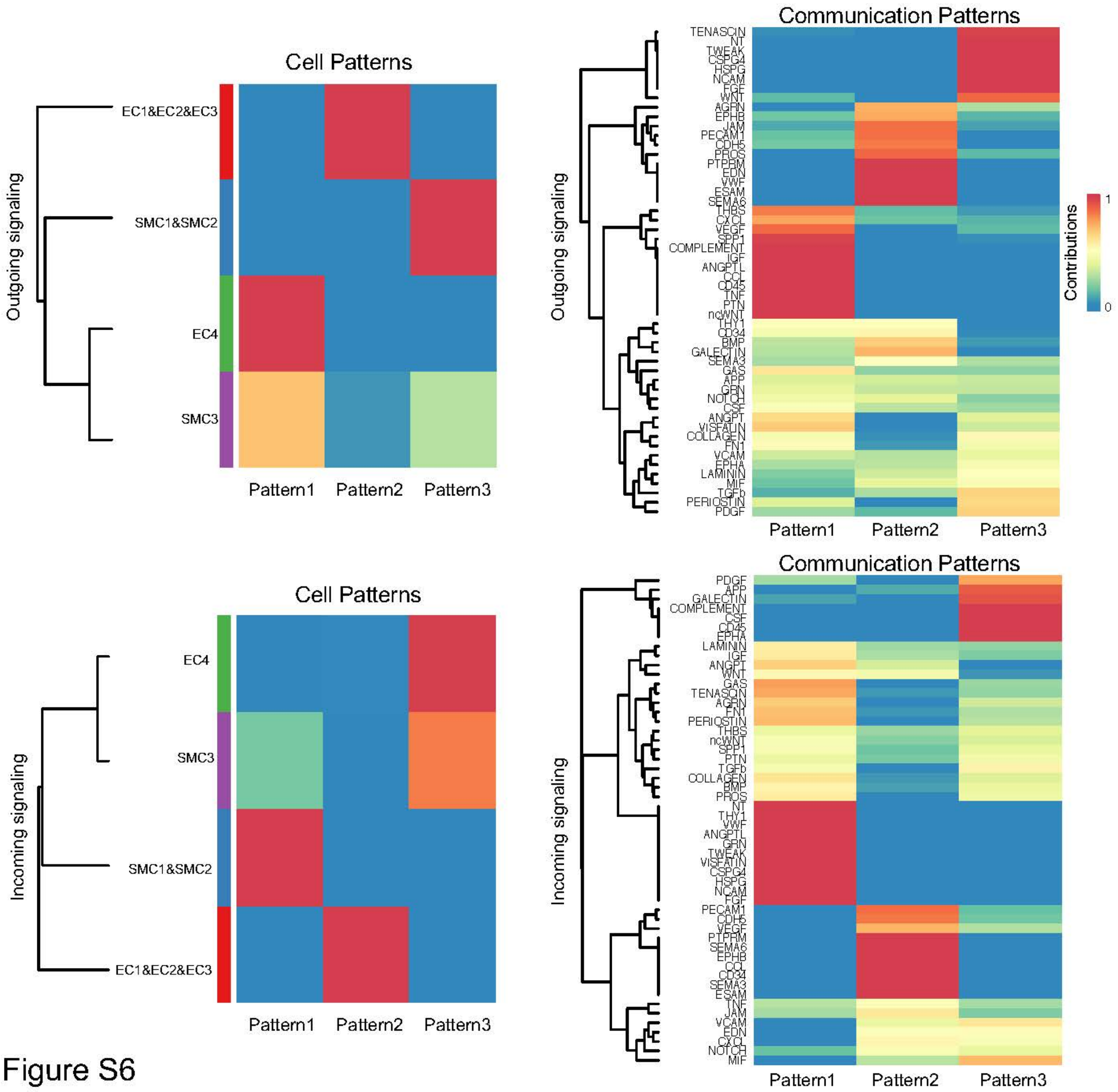

Figure S6

Predicted communication patterns of outgoing (top) and incoming (bottom) signaling pathways from all cell populations (left) and communication probability score of each signaling pathway in each pattern (right).

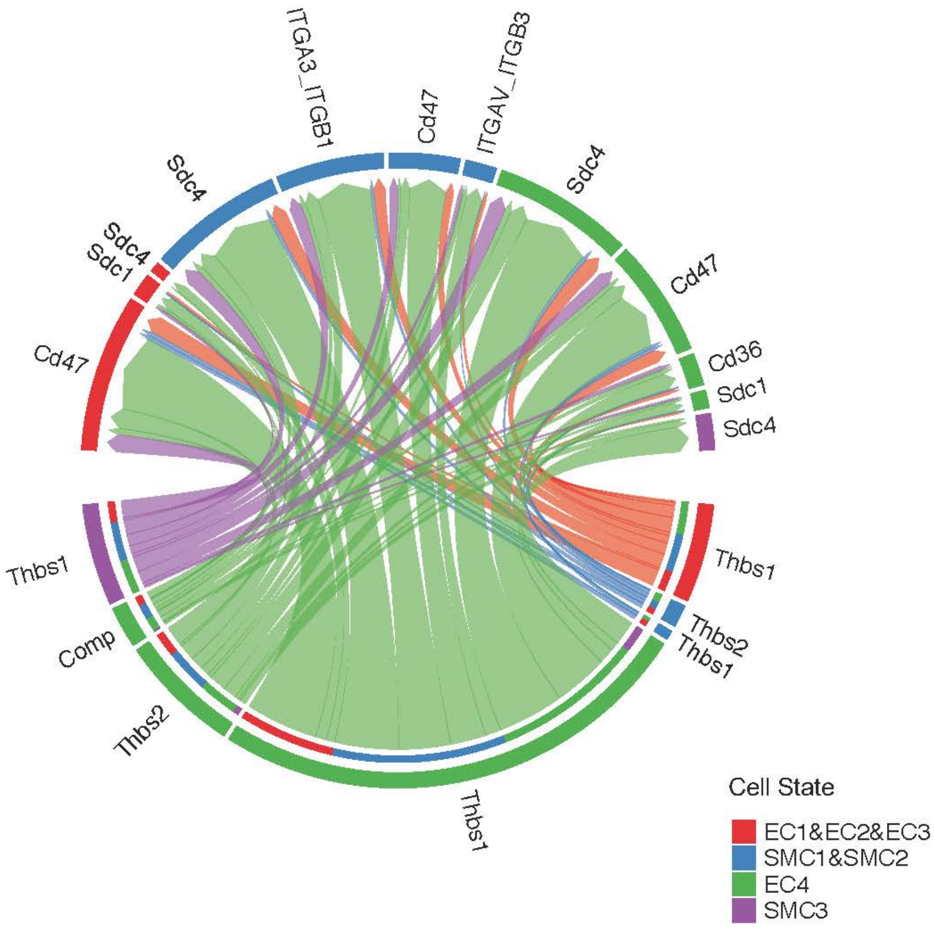

Figure S7

Chord diagram showing role of each sub-population in the Thbs signaling network with individual ligand and receptor pairing. The outside track indicates the sender groups and the inner track indicates the receiving groups.

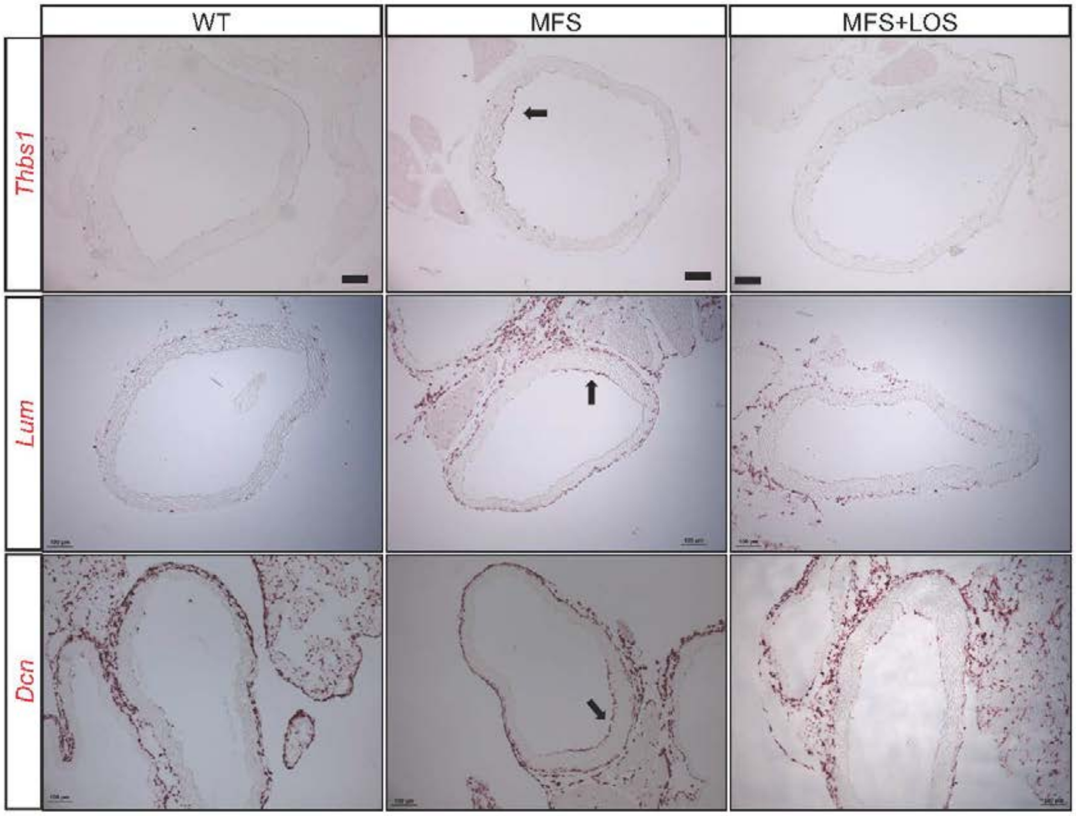

Figure S8

Representative images of aortic cross-sections from WT and *Fbn1*$^{mgR/mgR}$ (MFS) mice hybridized against the indicated probes. Scale bar = 100 µm.

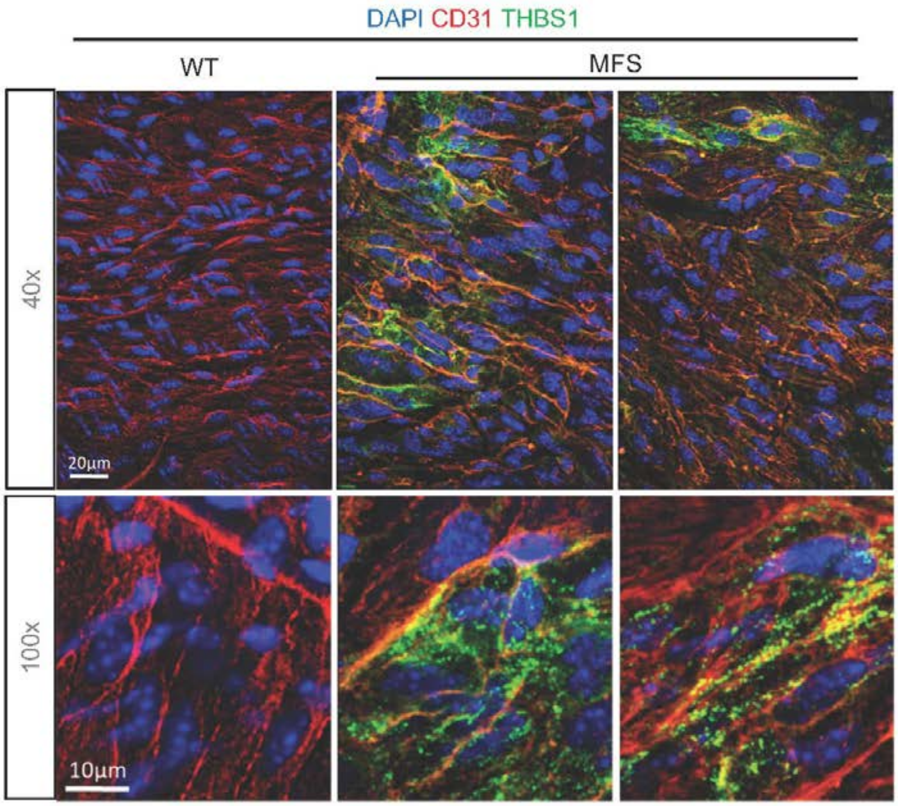

Figure S9

Illustrative images of *en face* aortic tissue sections from WT and *Fbn1*$^{mgR/mgR}$ (MFS) mice immunostained with CD31 (red) and THBS1 (green) antibodies, and DAPI (blue) counter-stained. Scale bars= 20 µm (top; 40X magnification) and 10 µm (bottom; 100X magnification).

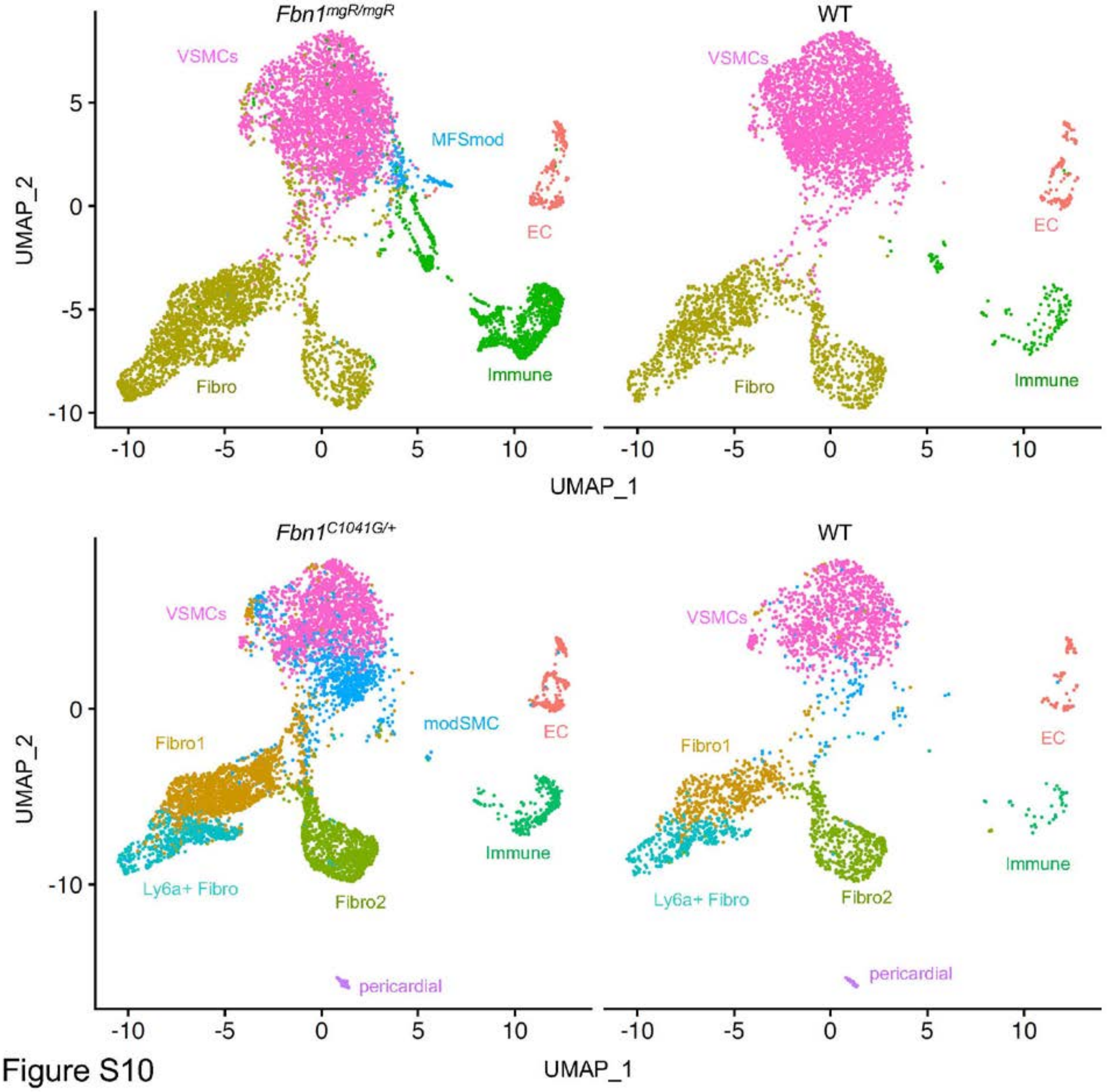

Figure S10

UMAP showing approximations of major aortic cell populations identified in *Fbn1*$^{mgR/mgR}$ and *Fbn1*$^{C1041G/+}$ mice (9). Of note, TAA-associated modSMC of *Fbn1*$^{C1041G/+}$ mice distribute in close proximity of TAA-associated MFSmod cells of *Fbn1*$^{mgR/mgR}$ mice. The upper panel shows the UMAP of *Fbn1*$^{mgR/mgR}$ mice and WT littermates with annotations used in this study, whereas the lower panel shows the counterparts of *Fbn1*$^{C1041G/+}$ mice and WT littermates with the same annotations as employed previously by Pedroza et al. (9).

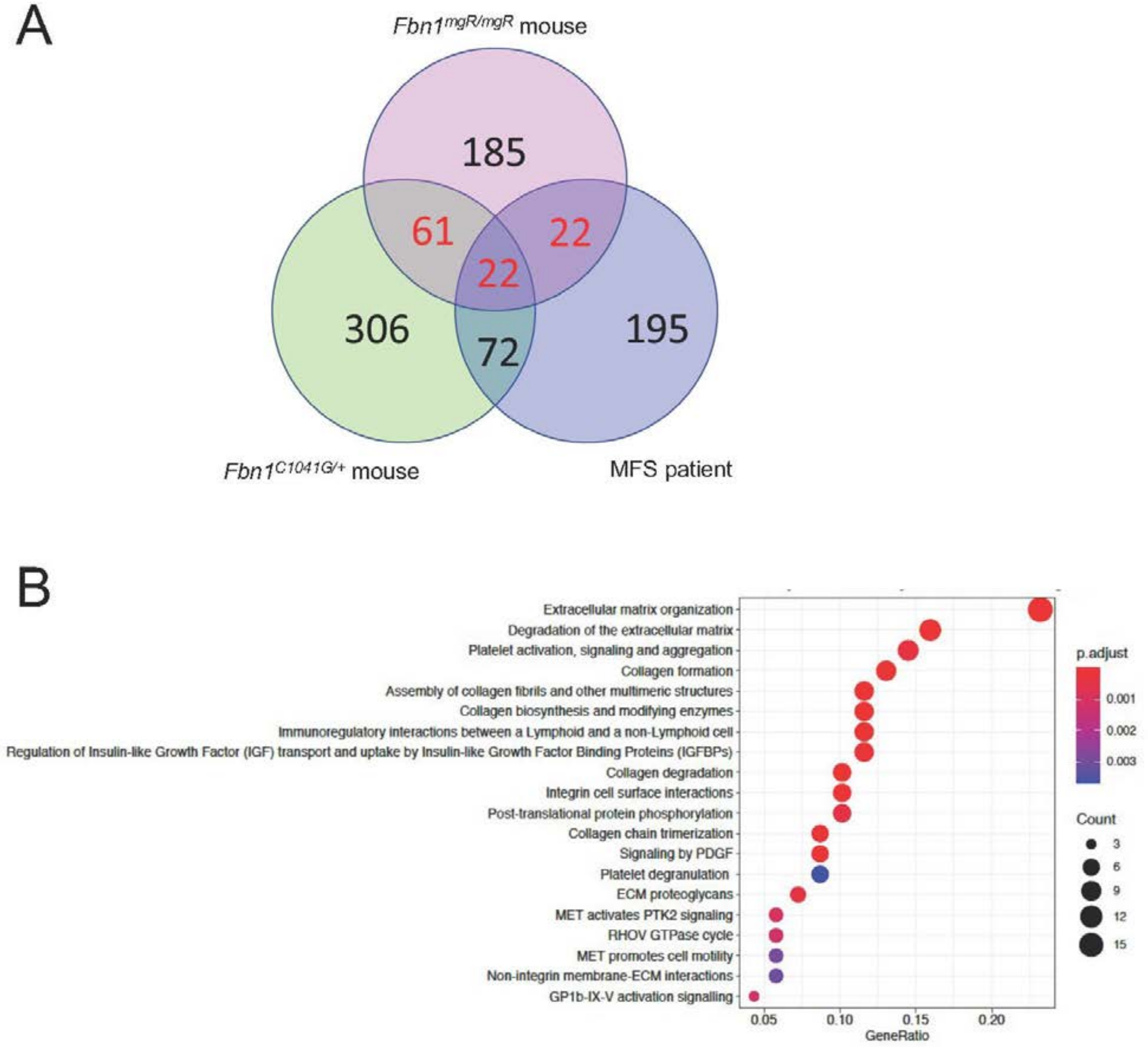

Figure S11

(**A**) Venn diagram showing the number of up-regulated genes in MFSmod (pink circle), and in mouse (green circle) and human (blue circle) modSMC (9) (adjusted *p* values <0.05, average log2 fold change >0.25 used as a threshold). Number of genes in common across the distinct disease models are highlighted in red. (**B**) Dot plots showing Reactome-based analysis of top 20 pathways enriched in the 105 genes shared among MFSmod of *Fbn1*$^{mgR/mgR}$ aortas and modSMC of *Fbn1*$^{C1041G/+}$ aortas and MFS patient's aortic root (9).

A

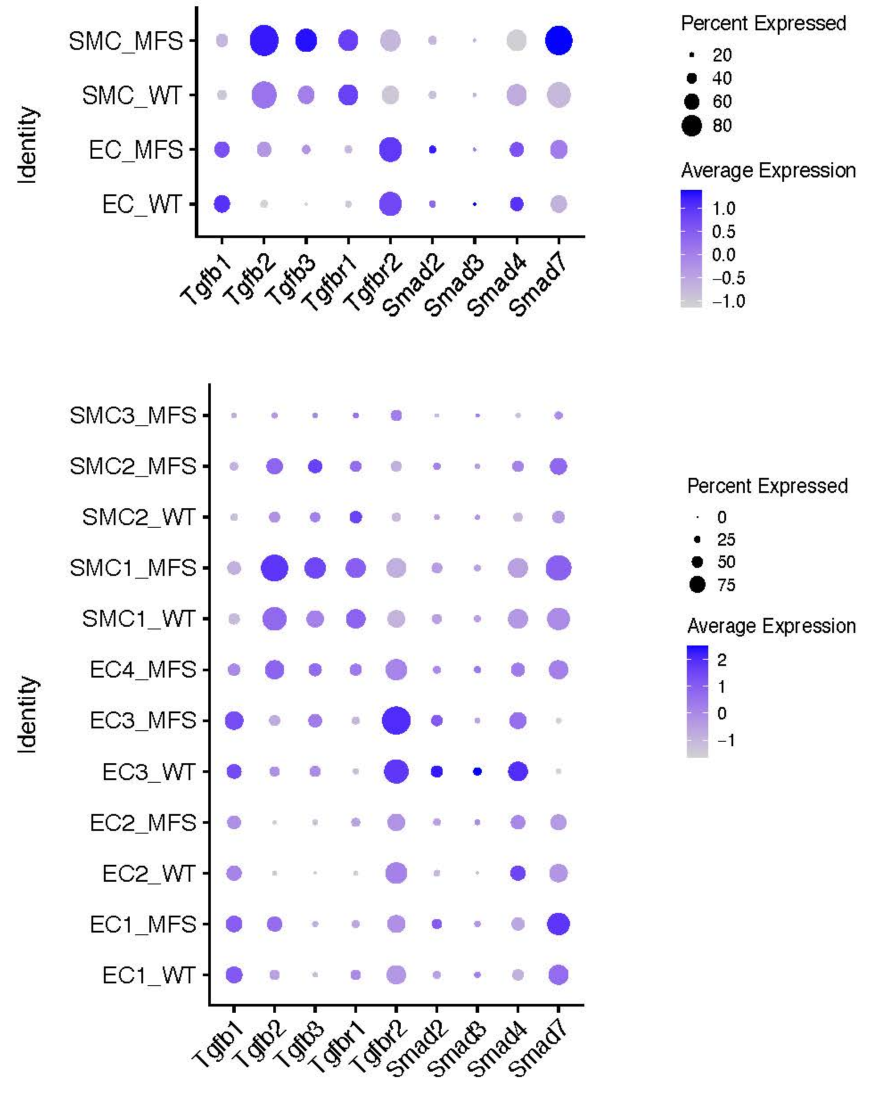

B

Figure S12

Dot plots showing expression of genes associated with the canonical TGFβ signaling pathway in the combined SMC and EC populations (**A**), and in each of the SMC and EC sub-populations (**B**) of *Fbn1*$^{mgR/mgR}$ mice. Dot size corresponds to the percentage of cells expressing each gene, and dot color corresponds to the level of expressions.
